# Saddle Thigh Block Design Can Influence Rider and Horse Biomechanics

**DOI:** 10.3390/ani13132127

**Published:** 2023-06-27

**Authors:** Rachel Murray, Mark Fisher, Vanessa Fairfax, Russell MacKechnie-Guire

**Affiliations:** 1 Rossdales Veterinary Surgeons, Newmarket, Suffolk CB8 7NN, UK; 2 Woolcroft Saddlery, Mays Lane, Wisbech PE13 5BU, UK; woolcroft2002@yahoo.co.uk; 3 Fairfax Saddles, The Saddlery, Fryers Road, Bloxwich, Walsall, West Midlands WS3 2XJ, UK; vanessa.fairfax@fairfaxsaddles.com; 4 Centaur Biomechanics, Dunstaffanage House, Moreton Morrell, Warwickshire CV35 9BD, UK; russell.mackechnie-guire@hartpury.ac.uk; 5 Department of Clinical Science and Services, The Royal Veterinary College, Hawkshead Lane, Brookman’s Park, Hatfield AL9 7TA, UK

**Keywords:** kinematics, dressage, equine, pressure

## Abstract

**Simple Summary:**

There is increasing interest in the effect of saddle design on horse kinematics, but little evidence of the influence on rider–saddle interaction and how this affects horse movement patterns. We aimed to investigate the effect of changing the design of the saddle’s thigh block on the interaction between the rider and saddle and the effect this has on rider movement and horse movement. To do this, we used a seat pressure mat between the rider and the saddle and tracking technology to analyse horse and rider movement. Elite level sports horses, ridden by elite level riders, were trotted in well-fitting dressage saddles that were identical, except for the thigh block design. During straight-line locomotion when in sitting trot, results showed that a thigh block with a more deformable face (thigh block F) resulted in a greater contact area and more pressure between the rider’s seat and the saddle as well as a more upright rider position when the horse’s limbs were on the ground. An association between thigh block design, horse spinal movement, and forelimb flexion was also seen. These findings illustrated the importance of optimizing rider–saddle–horse interaction.

**Abstract:**

The association between rider–saddle interaction and horse kinematics has been little studied. It was hypothesized that differences in a thigh block design would influence (a) rider–saddle interface pressures, (b) rider kinematics, and (c) equine limb/spinal kinematics. Eighteen elite sport horses/riders were trotted using correctly fitted dressage saddles with thigh blocks S (vertical face) and F (deformable face). Contact area, mean, and peak pressure between rider and saddle were determined using an on-saddle pressure mat. Spherical markers allowed for the measurement of horse/rider kinematics using two-dimensional video analysis. The kinematics of the equine thoracolumbosacral spine were obtained using skin-mounted inertial measuring units. Results were compared between thigh blocks (paired *t*-test *p* ≤ 0.05). With F, the contact area, mean, and peak pressure between rider and saddle were significantly higher (*p* = 0.0001), and the rider trunk anterior tilt was reduced, indicating altered rider–saddle interaction. The horse thoracic axial rotation and flexion/extension were reduced (*p* = 0.01–0.03), caudal thoracic and lumbar lateral bend was increased (*p* = 0.02–0.04), and carpal flexion increased (*p* = 0.01–0.05) with F compared to S. During straight-line locomotion when in sitting trot, thigh block F was associated with altered rider–saddle interaction and rider and equine kinematics, leading to a more consistent rider–saddle interface, a more upright rider trunk during stance, an increased horse thoracic stability and lumbar lateral bend, and forelimb flexion, supporting the importance of optimising rider–saddle–horse interaction.

## 1. Introduction

There have been significant advances in the understanding of the effect that saddle fit and design has on equine locomotion [1,2,3,4,5,6,7], but there has been less study of the influence of the saddle–rider interaction on rider kinematics and equine locomotion [8]. The saddle provides an interface between the rider and horse, aligning the rider’s centre of mass (CoM) with the horse’s CoM. A rider’s ability to perform a pelvic tilt has been reported to influence horse–rider synchronisation [9], and increased pelvic mobility and control resulted in fewer conflict behaviours [10]. Therefore, it is possible that a saddle design restricting the function of the rider could impact the interaction with the horse and potentially influence the horse to adopt a locomotor pattern to compensate [11].

In trot, there are two flexion/extension cycles per stride, and the horse’s diagonal limbs are synchronised, resulting in large vertical and longitudinal accelerations [12]. The rider’s axial body segments (pelvis, trunk, head) accommodate the translational and rotational movements of the horse’s trunk, which allows the arms and legs to function independently, applying precise aids to the horse [13]. For each stride cycle, during the first half of the diagonal stance phase, the axial segments alter, with the rider’s pelvis rotating anteriorly whilst the trunk rotates posteriorly, resulting in lordosis of the rider’s lumbar spine. The hip joint is flexed and abducted, and the rider’s thigh is flexed and adducted. During the second half of the horse diagonal stance phase, the segmental rotations are reversed [14].

The rider’s pelvis is the platform that supports the upper and lower segments and allows for effective weight distribution through the rider’s seat, applying subtle cues to the horse. The features of the rider-facing side of the saddle in relation to the rider segments are important considerations, as restrictions of any of the rider segments may compromise the effectiveness of the rider’s seat and affect the mobility and ability of the rider to effectively absorb the dynamic forces that occur during locomotion. Whilst the rider may still be able to direct the horse to perform the required tasks, the rider’s effectiveness or synchronicity with the horse may be compromised, which could impact the horse’s movement; thus, it is important to evaluate the effect that the rider’s compensatory strategies may have on the horse [15].

One of the features of the rider-facing side of the saddle is the knee block. Knee blocks are non-deformable, and their function is to support the rider’s knee, as the knee is resisted from travelling forwards during locomotion. Over the past decade, knee block size has increased, with the knee block evolving to support the thigh and being referred to as thigh blocks. Various thigh block designs are available in terms of shape, size, height, and position. A rider may influence a thigh block selection, with some riders preferring a larger thigh block, as it will provide additional support whilst riding, whereas others may feel that a larger thigh block is restrictive. Although thigh blocks are a prominent feature on the saddle, little is known about their effect on the rider and, consequently, the horse.

Therefore, it is possible that restriction of rider movement by a thigh block during the stride cycle might alter the rider’s movement patterns, the effectiveness of the seat, and their ability to move synchronously with the horse, which, in turn, has the potential to affect equine locomotion. The aim of this study was to investigate the effect that thigh block shape and design has on the kinematics of elite horses and riders during straight line locomotion when performing the sitting trot. It is hypothesised that the contact area and the magnitude of pressures between the rider’s seat and saddle, rider trunk and leg kinematics, and horse thoracolumbar and limb kinematics would differ between two different thigh block designs positioned on a standardised saddle: thigh block S (a conventional, vertical-faced solid block) and thigh block F (a block with a multi-layered deformable face).

## 2. Materials and Methods

Ethics and welfare committee approval was attained from the Royal Veterinary College and the Animal Health Trust committees (URN 2018 1785-2 and 14-2016, respectively). Before the study, riders provided informed consent using a standardized form. Riders and horses could be withdrawn at any stage in the study.

### 2.1. Horses

Eighteen elite sports horses (12 dressage and 6 event horses; thirteen geldings, four mares, and one stallion) were included in the study. They had a mean (± standard deviation) wither height of 1.65 ± 0.09 m, body mass of 595 ± 27 kg, and were aged 11 ± 1 years. Horses underwent regular therapy and veterinary assessments as part of their management programme and were assessed prior to the study. This assessment included veterinary visual observations when walking and trotting in a straight line and a physiotherapy examination by an Association of Chartered Physiotherapists in Animal Therapy chartered physiotherapist. On the day of data collection, the horses’ gait asymmetry was quantified using a validated sensor system [16]. Horses were included in the study if they had no lameness or orthopaedic problems and were deemed fit to perform upon the veterinary and physiotherapy examination. 

### 2.2. Riders

Two male and two female FEI Grand Prix Dressage and one male and one female FEI ranked five-star event riders were recruited with an average (±standard deviation) height 1.78 ± 0.06 m and body mass 71 ± 10 kg. All riders were healthy and uninjured.

### 2.3. Saddles, Girths and Bridles

Horses were ridden in their usual dressage saddle, girth, and bridle, which were under regular assessment and maintenance. On the day of the study, static and dynamic saddle fit to both rider and horse were assessed independently by five Society of Master Saddlers Qualified Saddle Fitters. The same model of dressage saddle, as described by Murray et al., 2017 [5], was used, with the only variation being the thigh block design. Thigh block S was a moulded block, 260 mm long, featuring a vertical face covered in leather with no additional padding. Thigh block F was 260 mm long; however, the rider-facing aspect of the thigh block was concave and layered with three closed-cell foams of varying densities to form a deformable face. For both the thigh block S and thigh block F conditions, saddle seat size and stirrup leather length remained the same throughout. An anatomically shaped girth not featuring any elastic was used throughout. Girth design and features have been described elsewhere [17]. All horses were ridden in an anatomically shaped snaffle bridle (Sprenger KK Ultra Snaffle Bit) with a fitted crank cavesson noseband located between the facial crest and the corner of the lips and fitted to a two-finger (index and middle finger) tightness measured between the midline of the nasal bone and noseband. All noseband tightness values were measured by the same research assistant. 

### 2.4. Rider Kinetics—Pliance Seat Mat

**Figure 1:**
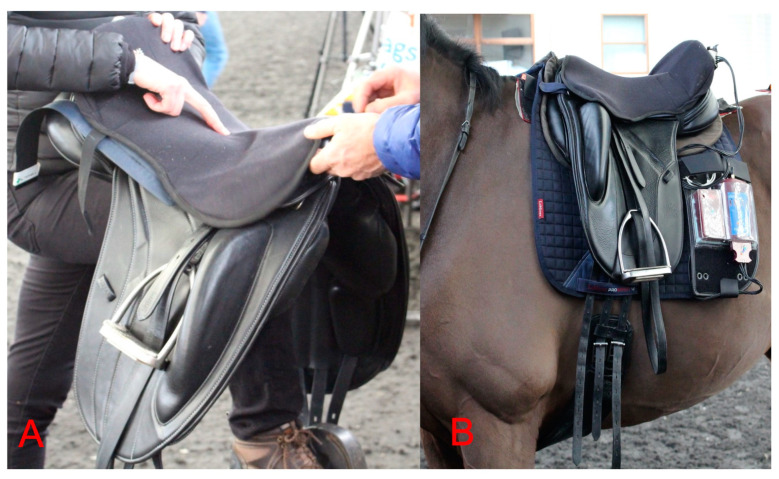
(**A**) Pliance force mat was positioned on top of the saddle beneath a thin cover, which was designed to prevent the force mat from displacing during locomotion. The force mat measured mean and peak pressures (kPa) with simultaneous video, and (**B**) the cables from the force mat were connected to a Pliance data logger, which was secured to the saddle pad.

A force mat was positioned on top of the saddle, quantifying the riders’ seat pressures (force per unit area), (sensor size: 10 × 10 mm^2^; mat dimensions: 160 mm long and 160 mm wide; sensor arrangement: 256 sensors arranged in 16 columns and 16 rows; pressure range: 2–600 kPa; and sensor resolution: 1 sensor per cm^2^) (Sensor Elastisens ES Mat S2129, seat saddle mat, Pliance, Novel gmbh, Munich, Germany) (sampling rate 50 Hz) (Figure 1). To ensure that the force mat did not displace during locomotion, the force mat was positioned within a thin cover. All riders were accustomed to the experimental cover and mat prior to testing. Bluetooth technology was used to capture force mat data. Video footage was simultaneously recorded (50 Hz Panasonic, Osaka, Japan). The mat was centralized and then initialized to zero at the start of the study, between each saddle measurement set and recalibrated during the study based on the manufacturer’s guidelines. Using the simultaneous video data (50 Hz) and seat pressure data, the point in the stride at which the peak pressures occurred in each thigh block type was determined. Contact area (cm^2^), mean, and peak (kPa) pressure data were obtained for the sensors loaded in the region of the rider’s seat bones (tubera ischii).

### 2.5. Kinematics—Inertial Measurement Units 

Seven inertial measurement units (IMU) (aXsens) were used, either attached to each horse’s skin/hair surface using hair extension glue (Salon Pro), located over the fifth thoracic vertebra (T5) (withers); T13; and third lumbar vertebra (L3); or in custom-made pouches over the occiput (poll) and on the dorsal midline at the level of the tubera sacrale (TS), attached with double-sided tape. These were used as part of a sensor-based system (Xsens MTw Awinda), which has been validated for translational displacements derived from internal tri-axial sensor accelerations, which were then rotated into a horse-based reference frame based on the sensor orientation estimate and then double integrated into the displacement [16,18]. Data processing methods have been described elsewhere [19,20]. In brief, orientation-time signals for differential axial rotation, flexion-extension, and lateral bending values of T5, T13, L3, and TS were used to calculate differential rotational movement, as described by MacKechnie-Guire and Pfau 2021a, 2021b [2,19]. 

### 2.6. Two-Dimensional Motion Capture 

**Figure 2:**
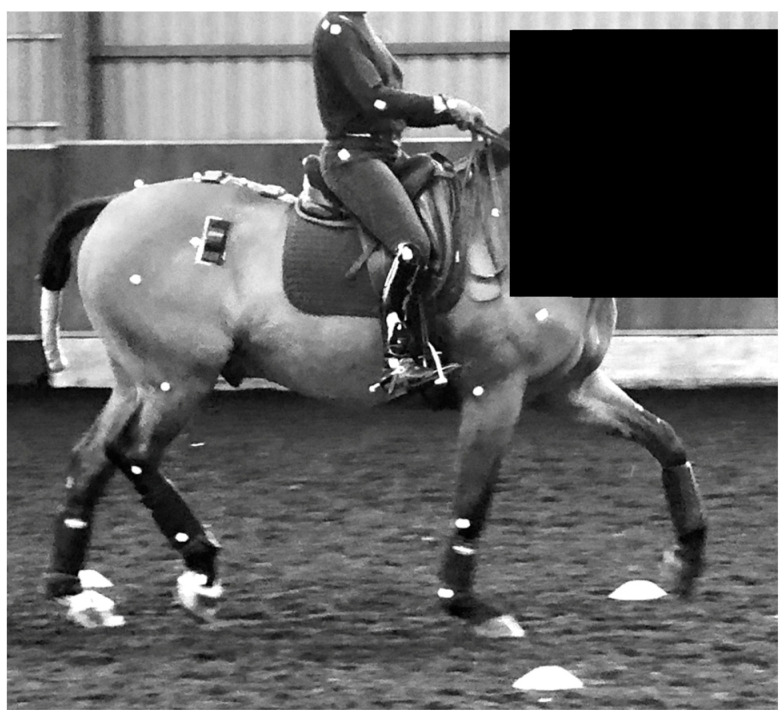
Illustrating marker locations for the horse and rider used for two-dimensional motion capture to compare horse and rider kinematic data between two different thigh block designs.

Kinematic data were recorded with a high-speed video camera system, using 24 skin markers (30 mm; Quintic Consultancy, West Midlands, UK) placed on each horse using double-sided tape. Marker locations were identified by manual palpation of anatomical landmarks identifying joint centres and segment ends. Once located, white skin paint was used to mark each reference point. Markers were located on (1) dorsal extent of scapular spine, (2) greater tubercle of humerus, (3) lateral epicondyle of humerus, (4) proximal extent of the fourth metacarpal bone, (5) lateral condyle of the third metacarpal bone, (6) tuber coxae, (7) greater trochanter of the femur, (8) lateral epicondyle of the femur, (9) talus, (10) lateral condyle of the third metatarsal bone, and (11) origin of the LCL of the distal interphalangeal joint (Figure 2). 

Rider kinematics in relation to the horse were quantified by applying 30 mm spherical markers on anatomical landmarks. Markers were positioned on anatomical landmarks, illustrating marker location for the rider: (1) lateral aspect of the proximal humerus, (2) lateral epicondyle of the distal humerus (3) lateral aspect of the radiocarpal joint, (4) lateral aspect of the greater trochanter of the femur, (5) lateral aspect of the proximal extent of the fibula, (6) lateral aspect of the distal extent of the fibula (Figure 2). Markers were fitted and checked between trials by the same chartered physiotherapist from the Association of Chartered Physiotherapists in Animal Therapy. To limit the effect that clothing had on marker position, riders wore fitted base layers. 

One high-speed camera (Quintic, Coventry, United Kingdom) was positioned at a 10 m distance from the experimental track, capturing one side of the horse and rider at 300 Hz (spatial resolution: 1300 × 400, 300 fps, at 10 m distance), with a field of view capturing two complete strides in trot. High-speed video data were recorded and downloaded to a laptop (Lenovo, Hong Kong, China) and processed using two-dimensional motion capture (Quintic Biomechanics, Quintic Consultancy, West Midlands, UK). Automatic marker tracking was used to investigate equine limb and rider kinematics.

### 2.7. Experimental Protocol

**Figure 3:**
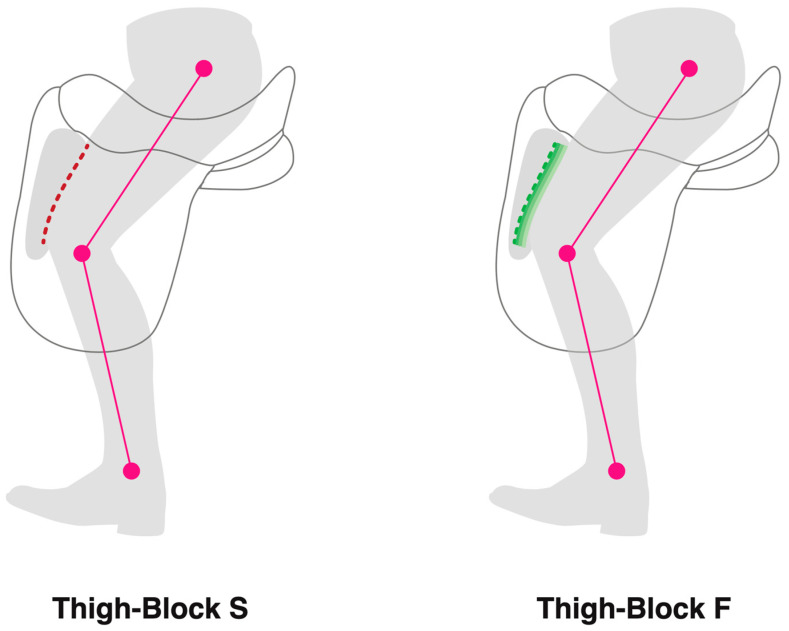
Illustrating thigh block S, a moulded block 260 mm long featuring a vertical face covered in leather with no additional padding (red dashed lines). Thigh block F was also 260 mm long; however, the rider-facing aspect of the thigh block was concave and layered with three closed-cell foams of varying densities to form a deformable face with the most deformable layer facing the rider (three shaded green lines). Markers located on the lateral aspect of the greater trochanter of the femur, the lateral aspect of the proximal extent of the fibula, and the lateral aspect of the distal extent of the fibula.

Horses were ridden in matching saddles (dressage monoflap saddle, 17 ½” seat size), with either thigh block S (a conventional vertical thigh block) or thigh block F (a multi-layered deformable-face thigh block) (Figure 3) in a randomized order (stratified randomization) with identical girth, saddle cloth, and half pad, as described by Murray et al., 2017 [5] Data were collected from half of the studied horses when they were fitted with a saddle, featuring thigh block S first followed by thigh block F second, and the remaining horses were ridden first with a saddle, featuring thigh block F first and second with thigh block S. Each horse underwent a 15 min warm up, including walk, rising/sitting trot, and canter on both left and right reins, as prescribed by the rider. After the warm-up period had been completed, the rider’s seat kinetics and body kinematics were quantified along with the kinematics of the thoracolumbar spine and limbs during straight line locomotion in sitting trot. 

An experimental area (50 m × 1.5 m) was created using spherical cones to define a straight line in an indoor (20 m × 60 m) arena, with electronic timing gates marking the start and end points, which were used to define speed. Data were collected from the straight-line experimental area, with the horse moving through the arena in clockwise (2 repeats) and anti-clockwise (2 repeats) directions in sitting trot, and the arena dimensions allowed for eleven repeated straight-line strides to be captured. 

### 2.8. Data Outcomes

**Figure 4:**
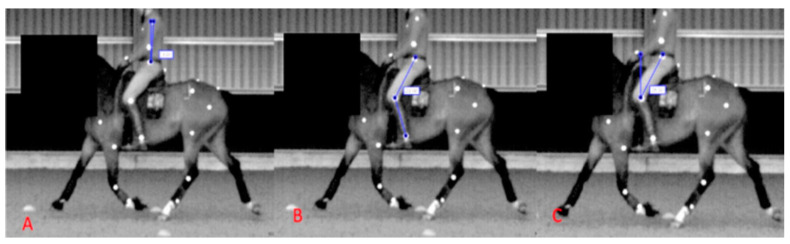
Illustrating the angles measured when quantifying rider kinematics: (**A**) = rider’s trunk angle relative to the vertical, (**B**) = rider’s knee angle, and (**C**) = rider’s femur angle relative to the vertical. Rider kinematics were obtained for each stride point on both the left and right rein.

For both conditions (thigh block S and thigh block F), kinetic (Pliance) and kinematic (IMU) data were obtained from a total of 22 ± 2 straight-line strides, and kinematic (two-dimensional video analysis) data were obtained from a total of 4 ± 1 strides (from both a clockwise/anticlockwise approach) included in the analysis. For the rider outcome parameters, mean and peak seat pressures (kPa) were quantified for 22 ± 2 straight-line strides, and the rider’s trunk angle (Figure 4A), femur angle relative to the vertical (Figure 4C), and knee angle (Figure 4B) were quantified at three stride points (point of contact, midstance, and last point of contact). Outcome parameters for the IMU-derived data were flexion-extension, axial rotation, and lateral bending differential values for T5-T13, T13-L3, and L3-TS. For the two-dimensional video analysis, outcome parameters were maximum shoulder, elbow, carpal, hip, stifle, and tarsal flexion during the swing phase.

### 2.9. Data Analysis

Descriptive data analysis was performed to investigate the data. A Shapiro–Wilks normality test was used to determine data distribution. Paired Student’s *t*-test (for parametric data) or Wilcoxon sign rank test (for nonparametric data) were performed to compare rider contact area and seat pressures, rider kinematics, and equine thoracolumbar and limb kinematics between thigh blocks S and F for each horse. All analyses were performed using statistical analysis software (Analyse-It for Microsoft Excel version 3), with a significance level of *p* ≤ 0.05. 

## 3. Results

### 3.1. Horse Inclusion

All horses were deemed fit to perform based on the subjective veterinary and physiotherapy assessments. No horses had hypertonicity or pain in the thoracolumbosacral epaxial musculature. From the objective movement asymmetry measures, the horses had (mean ± SD) asymmetry values (in mm): Head MinDiff 7.3 ± 5.7, Head MaxDiff −4.0 ± 2.5, Pelvis MinDiff, 2.1 ± 2.2, Pelvis MaxDiff 3.1 ± 2.6, and HHD 4.9 ± 4.3.

#### Rider Seat Pressures (kPa) and Contact Area (cm^2^)

**Figure 5:**
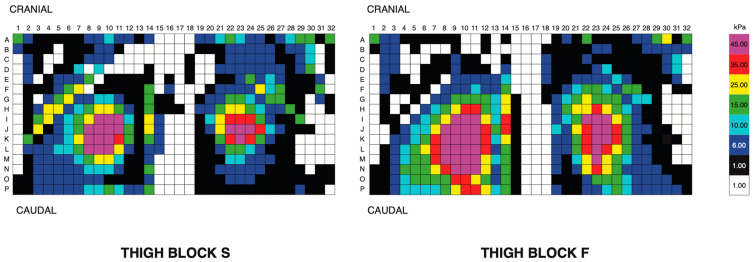
Pressure distribution between rider seat and saddle from one rider (dressage) using thigh block S (**left**) and thigh block F (**right**). Mean and peak pressures were greater and located further caudally with thigh block F than with thigh block S.

The maximum mean and peak pressures occurred during 75–80% of the diagonal stance phase. The differences in the seat pressure magnitude (kPa) and positioning were found between thigh blocks. Thigh block F was associated with greater mean pressures (*p* ≤ 0.0001) (thigh block S, 6.2 ± 1.7 kPa; thigh block F, 7.6 ± 2.5 kPa) and peak pressures (*p* ≤ 0.0001) (thigh block S, 13.5 ± 3.9 kPa; thigh block F, 15.7 ± 4.4 kPa) than thigh block S (Figure 5).

Thigh block F was associated with significantly greater total contact area between the rider’s seat and saddle than thigh block S (*p* = 0.0003) (thigh block S, 401.4 ± 76.5; thigh block F 430.2 ± 65.8), with thigh block F having a 7.2% higher contact area than S. This pattern remained present when the contact area of the cranial and caudal halves of the saddle seat were compared separately with thigh block F, having a 5.5% greater contact area in the cranial half and a 9.1% higher contact area in the caudal half of the saddle (cranial: thigh block S, 211.4 ± 32.1; thigh block F, 223.0 ± 26.0. Caudal: thigh block S, 190.1 ± 53.4; thigh block F, 207.2 ± 47.2). 

### 3.2. Rider Kinematics 

Differences in rider trunk kinematics were found between thigh blocks at mid-stance. During mid-stance, the rider’s trunk had less posterior tilt and was more vertical (closer to zero) with thigh block F compared to thigh block S (left rein: thigh block S, 3.4° ± 3.4°; thigh block F, 1.9° ± 2.1°; *p* = 0.009. Right rein: thigh block S, 4.2° ± 4.5°; thigh block F, 2.0° ± 2.9°; *p* = 0.003). No differences were found in the remaining rider-based parameters (All > *p* = 0.06) (Table 1). 

### 3.3. Equine Thoracolumbosacral Spinal Movement

#### 3.3.1. T5-T13

Flexion extension (thigh block S, 9.0° ± 3.6°; thigh block F, 7.3° ± 1.7°; *p* = 0.03) and axial rotation (thigh block S, 18.3° ± 5.8°; thigh block F, 15.6° ± 3.6°; *p* = 0.03) were significantly less for thigh block F than S. No difference in lateral bending values were detected (*p* ≥ 0.38). 

#### 3.3.2. T13-L3

No differences in flexion/extension were found between thigh blocks (*p* ≥ 0.20), but differences were observed in axial rotation and lateral bending. Thigh block F was associated with less axial rotation (thigh block S, 14.6° ± 5.0°; thigh block F 12.4° ± 3.1°; *p* = 0.02) and greater lateral bending (thigh block S 8.6° ± 2.4°; thigh block F 9.4° ± 1.9°; *p* = 0.03) than thigh block S.

#### 3.3.3. L3-TS

No differences between conditions were found in any differential parameters (all *p* ≥ 0.43).

### 3.4. Limb Kinematics (Swing Phase) (°)

Maximum carpal flexion differed between conditions (smaller value = increased flexion). Thigh block F was associated with significantly greater carpal flexion than thigh block S (thigh block S, 91.4° ± 8.2°; thigh block F, 90.3° ± 8.2°; *p* = 0.05). No differences were found between conditions for the remaining limb kinematic parameters (all *p* ≥ 0.54) (Table 2).

## 4. Discussion

The aim of this study was to investigate the differences in rider kinematics, pressures between the rider’s seat and the saddle, and horse thoracolumbosacral and limb kinematics between a dressage saddle with two different thigh block designs: a conventional vertical-faced block (thigh block S) and a multi-layered deformable block (thigh block F) when in sitting trot during straight-line locomotion. In accordance with our experimental hypothesis, different thigh block designs were associated with differences in rider and horse kinematics. Thigh block F was associated with more vertical orientations of the rider’s trunk during mid-stance and greater pressures between the rider’s seat and the saddle, alongside greater flexion/extension and axial rotation of the horse’s cranial thoracic spine, greater axial rotation and less lateral bend in the caudal thoracic–lumbar spine, and an increase in swing phase peak carpal flexion compared to thigh block S.

Thigh blocks on saddles tend to be made from a hand-rasped block of a semi-rigid material (such as closed-cell polyethylene foam) or moulded from polyurethane foams of varying densities. This means thigh block design and shape varies radically in width, height, and length and from one model of saddle to another. How the block interfaces with the rider’s thigh in motion can also be influenced by many factors such as the location of the block on the saddle, the location of the stirrup bar, the sweep of the seat, the density of the seat, as well as how the saddle is fitted and balanced on the horse. Therefore, we considered it important to ensure that all these features were identical between saddles, except for the thigh block design. In our study, these variables were controlled, and only the thigh block design was different between conditions. Thigh block S was a conventional moulded design with a vertical face against the rider’s thigh, and this was compared with a unique design of a moulded block, which had a concave surface, which allowed a deformable multi-layered foam face to be incorporated against the rider’s thigh (thigh block F). By only changing this one feature of the saddle, we were able to assess the effect this change had on the interface between the rider, saddle, and horse in motion.

The timing of the peak pressures beneath the rider’s seat occurred during 75–80% of the diagonal stance phase of the horse stride. This part of the stride coincided with large acceleration forces, as the diagonal pair of limbs generated propulsive forces to raise the horse’s trunk dorsally and cranially into suspension. During this phase of the stride, it has been reported that the rider’s pelvis rotates posteriorly, the rider’s trunk rotates anteriorly, and the hip joints extend and are adducted whilst the knee extends and is abducted [14]. 

The magnitude of mean and peak (kPa) seat pressures were greater, and the seat contact area was greater for thigh block F, which could potentially be related to the relative orientation of the rider’s trunk, which had less anterior tilt throughout the horse stance phase in thigh block F. The axial segments work cohesively and are influenced by the pelvis. Therefore, if the rider’s trunk is more vertical as a function of the thigh block design, it seems reasonable to expect that the remaining segments may be altered. The findings presented here, concerning the rider-facing saddle features influencing rider kinematics, are supported by a study where saddle flaps were removed. In that study, when walking, trotting, and cantering, the rider’s centre of pressure (CoP) was reduced in a medio-lateral direction and in an anteroposterior direction when performing a collected trot, extended trot, and extended canter with a flapless saddle. It was suggested that the rider’s femoral segments being positioned in a more adducted position relative to the horse could provide increased stability to the rider [8]. This change in rider CoP did not alter horse stride length, however, and more detailed locomotor parameters were not reported. 

We found that the flexion-extension and axial rotation values of the cranial thoracic spine (T5-T13) were decreased whilst lateral bending values were increased in the mid-thoracic and cranial lumbar spine (T13-L3) compared with thigh block S when riding in a dressage saddle with the multi-layered thigh block (thigh block F). This suggested that alterations in the rider–saddle interface could be having an impact on the horse, potentially altering the stability of the equine cranial thoracic spine. This concept was supported by a previous over-ground study quantifying back movement in horses trotting (unloaded) compared with a rider (loaded), where it was reported that axial rotation and lateral bending rotational values of the cranial thoracic (T5-T13) spine decreased whilst the kinematics of the caudal thoracic and lumber spine (T18-L3) were increased when ridden (loaded) [19,20]. It was proposed by the authors that this decrease in movement amplitude in the cranial thoracic spine may have been indicative of an attempted “stability” mechanism, in order to withstand the dynamic forces of the rider [21] (and saddle) and more efficiently transmit dynamic forces from the forelimb (and head and neck) to the cranial region of the thoracic spine. Applying this stability concept to the current study suggested that altered rider kinematics could have been having an effect on the equine locomotor apparatus, the stability of the cranial thoracic region in particular. 

During the stance phase, the range of motion of the rider’s trunk was more vertical when riding in thigh block F. It was proposed that the rider was able to maintain a more stable trunk position during the stance phase with thigh block F, and, as a result, this may have exerted a stabilizing effect on the horse’s cranial thoracic region. In contrast, if the rider’s trunk had increased its anterior–posterior trunk rotation, it was hypothesized that this could have induced instability in the horse, which could have explained the increased rotational movement of the cranial thoracic spine, as seen with thigh block S. More work is needed to confirm this concept, but this study did provide further evidence for the importance of considering the effect that the upper-side’s saddle features could have on the rider–saddle interface and rider–horse interaction. It should be noted that a relatively high SD was found for the rider trunk data. This variation may have been indicative of individual rider conformation or trunk biomechanical strategies when riding. It is possible that these findings would be less applicable in less skilled riders who have less musculoskeletal strength and coordination, as suggested by findings in a previous study, where riders with less pelvic control were less synchronized with the horse [9]. In our study, we found effects of altering thigh block design on the horse and rider; thus, it is possible that altering other aspects of saddle design and, therefore, the rider–saddle interface, could also impact the rider biomechanics and potentially those of the horse. Further investigation of other features with less skilled horses/riders would be of interest.

This study did have limitations. Unfortunately, due to technical issues, data relating to the rider’s pelvic kinematics were omitted from the analysis. If the study were to be repeated, quantifying rider pelvic kinematics would be useful. Some of the differences being reported here were small and, although statistically significant, may have resulted from biological variation; therefore, caution should be applied when interpreting the findings being presented. However, the only modification to the dressage saddle was the thigh block face with the deformable layers, with all the remaining upper and underside saddle features (the sweep of the seat, saddle tree, seat design/size, fit) remaining the same between thigh blocks; thus, we considered it reasonable that only small differences would be found.

We quantified the horses’ back movements with the use of skin mounted IMU’s, and it is appreciated that these did not directly correlate to the centre of rotation of the vertebral body, as seen in more invasive approaches [22,23], which would have had significant ethical issues. The use of IMUs to quantify back movement has been validated [18,24] for quantifying back movement during in-hand locomotion. However, it is acknowledged that no studies have validated the use of IMUs during ridden conditions. Whilst the IMUs did not contact the saddle at any point during motion and were not removed when quantifying the two experimental conditions, adding the saddle and girth may have affected the displacement of the skin and, consequently, the sensor–skin interaction. An over-ground study using IMUs positioned along the midline of the back to quantify differences in rotational movement of the back compared trotting in-hand with no saddle to horses fitted with a saddle and girth. It was reported that axial rotation in the cranial thoracic region (T5) was reduced, whereas lateral bending was increased in the mid-thoracic and lumbar regions (T13-T18 and T18-L3), the findings of which suggested that the saddle and girth could alter axial rotational values (or reduce the magnitude of skin displacement) in the cranial thoracic region [25]. Using an IMU-based approach [19,20,26,27] to compare back kinematics of horses trotting in hand and when ridden in sitting trot, similar to the aforementioned study, the axial rotation and lateral bending were reduced in the cranial thoracic region (T5-T13). However, unlike the previous in-hand study [25], axial rotation was increased in the mid-caudal segments, which may have been due to the dynamic effect of the rider. Whilst the IMUs provided a non-invasive approach to quantifying back movement, the limitations should be considered when interpreting the data presented here.

To reduce rider variables [11], only elite riders were studied, and the horses were ridden in a frame that was consistent for the level of work (with the dorsal aspect of the horse’s head close to vertical); it is appreciated that the results may not be transferable to less-skilled riders and horses. Defining the horse’s frame may have influenced the segmental strategy used by the riders and, consequently, the seat pressures. We chose to quantify the effect that the thigh block face had on the rider and locomotor parameters with the rider remaining seated throughout the trot cycle, which would have influenced the rider’s posture. It is appreciated that different riding positions could have an effect on equine locomotion [28] and that the different riding positions may be influenced by the thigh block design. Therefore, the findings being presented here cannot be applied for all riding positions. This study quantified the immediate effects that a thigh block had on elite riders riding advanced dressage and event horses when in sitting trot during straight line locomotion. Therefore, a longitudinal study would be advantageous to determine if the differences being reported here were sustained or altered. Finally, this study only quantified horse and rider kinematics when trotting; future studies should quantify the effect that a saddle thigh block has on the rider–saddle–horse interaction when in walk and canter [29], when riding in different riding positions [28], and when used by less skilled or symmetrical riders [15].

## 5. Conclusions

Changing thigh block design in a dressage saddle with skilled riders was associated with altered rider kinematics, rider–saddle interactions, and equine kinematics in sitting trots in a straight line. An altered rider–saddle interface in a less restrictive thigh block was associated with greater horse thoracic stability and increased carpal flexion, supporting the importance of optimising the rider–saddle–horse interface.

## Figures and Tables

**Table 1:** Rider kinematics parameters (mean ± SD) for the first point of contact, mid-stance, and last point of contact during the trot motion cycle for thigh block S and thigh block F assessed from the left (on the left rein) and right (on the right rein) sides when ridden in a straight line in identical saddles. Significant differences are shown in bold.

	Thigh Block S Left Trot Mean ± S.D	Thigh Block F Left Trot Mean ± S.D	*p* Value	Thigh Block S Right Trot Mean ± S.D	Thigh Block F Right Trot Mean ± S.D	*p* Value
**Rider’s trunk angle relative to the vertical**						
First Point of Ground Contact (°)	4.9 ± 4.2	4.6 ± 3.6	0.48	6.8 ± 6.4	5.7 ± 4.8	0.24
Mid-stance (°)	**3.4 ± 3.4**	**1.9 ± 2.1**	**0.009**	**4.2 ± 4.5**	**2.0 ± 2.9**	**0.003**
Last Point of Ground Contact (°)	6.1 ± 7.1	5.6 ± 6.6	0.54	5.3 ± 5.3	4.0 ± 3.9	0.06
**Rider’s thigh angle relative to the vertical**						
First Point of Ground Contact (°)	137.9 ± 4.3	138.4 ± 2.8	0.37	137.6 ± 1.9	136.8 ± 1.4	0.28
Mid-stance (°)	135.9 ± 3.8	135.3 ± 3.3	0.79	135.7 ± 4.1	134.5 ± 2.8	0.45
Last Point of Ground Contact (°)	138.4 ± 2.4	138.1 ± 2.5	0.59	139.6 ± 4.5	139.2 ± 3.3	0.80
**Rider’s femur angle relative to the vertical**						
First Point of Ground Contact (°)	23.8 ± 2.9	23.8 ± 2.7	0.50	26.9 ± 3.1	26.7 ± 3.7	0.81
Mid-stance (°)	28.4 ± 2.7	27.3 ± 4.7	0.29	27.8 ± 4.5	28.3 ± 4.6	0.64
Last Point of Ground Contact (°)	26.8 ± 3.3	26.6 ± 3.9	0.82	27.6 ± 4.7	28.3 ± 4.9	0.64

**Table 2:** Limb joint angles (mean ± SD) at peak flexion during the swing phase for horses ridden with thigh block S and thigh block F assessed from the left (on the left rein) and right (on the right rein) sides when ridden in sitting trot. Note: flexion is greater when joint angle is less.

	Thigh Block S			Thigh Block F			
	Trot Left Rein (Mean ± SD)	Trot Right Rein (Mean ± SD)	Trot Pooled (Mean ± SD)	Trot Left Rein (Mean ± SD)	Trot Right Rein (Mean ± SD)	Trot Pooled (Mean ± SD)	*p* Value (Left vs. Right Pooled)
Shoulder (°)	131.8 ± 38.0	129.1 ± 32.1	130.4 ± 43.2	112.1 ± 18.8	119.3 ± 7.1	115.5 ± 14.6	0.07
Elbow (°)	100.7 ± 4.7	99.9 ± 5.1	100.3 ± 4.9	100.1 ± 4.7	98.8 ± 5.5	99.5 ± 5.5	0.19
Carpal (°)	89.8 ± 8.2	92.6 ± 8.2	91.4 ± 8.2	89.9 ± 9.0	91.5 ± 7.5	90.3 ± 8.2	**0.05**
Hip (°)	100.1 ± 10.7	99.4 ± 7.5	99.7 ± 9.1	97.6 ± 7.8	98.9 ± 7.7	98.2 ± 7.7	0.06
Stifle Flexion (°)	122.8 ± 27.6	126.9 ± 22.1	124.9 ± 24.7	120.2 ± 17.3	125.2 ± 19.3	122.7 ± 18.3	0.21
Tarsal Flexion (°)	116.2 ± 7.1	118.3 ± 7.3	117.3 ± 7.2	116.5 ± 8.2	117.6 ± 7.5	117.1± 7.8	0.33

## Data Availability

Data are restricted for confidentiality reasons, due to the calibre of horses assessed.
